# Molecular Analysis of East African Lumpy Skin Disease Viruses Reveals a Mixed Isolate with Features of Both Vaccine and Field Isolates

**DOI:** 10.3390/microorganisms9061142

**Published:** 2021-05-26

**Authors:** Tesfaye Rufael Chibssa, Melaku Sombo, Jacqueline Kasiiti Lichoti, Tajelser Idris Badri Adam, Yang Liu, Yazeed Abd Elraouf, Reingard Grabherr, Tirumala Bharani K. Settypalli, Francisco J. Berguido, Angelika Loitsch, Mesfin Sahle, Giovanni Cattoli, Adama Diallo, Charles Euloge Lamien

**Affiliations:** 1 Animal Production and Health Laboratory, Joint FAO/IAEA Division of Nuclear Techniques in Food and Agriculture, Department of Nuclear Sciences and Applications, International Atomic Energy Agency, Friedenstrasse 1, A-2444 Seibersdorf, Austria; chibssasafo@gmail.com (T.R.C.); lydialiu329@hotmail.com (Y.L.); T.B.K.Settypalli@iaea.org (T.B.K.S.); F.Berguido@iaea.org (F.J.B.); G.Cattoli@iaea.org (G.C.); 2 National Animal Health Diagnostic and Investigation Centre (NAHDIC), Sebeta P.O. Box 04, Ethiopia; sombomelaku@yahoo.com (M.S.); sebeta.mesfin@gmail.com (M.S); 3 Institute of Biotechnology, University of Natural Resources and Life Sciences (BOKU), Muthgasse 18, A-1190 Vienna, Austria; reingard.grabherr@boku.ac.at; 4 Directorate of Veterinary Services, Ministry of Agriculture, Livestock and Fisheries, Private Bag, Nairobi 00625, Kenya; kasiiti.orengo@gmail.com; 5 Central Veterinary Research Laboratories, Animal Resources Research Corporation, Ministry of Livestock, Fisheries and Ranges, Khartoum P.O. Box 8067, Sudan; tajooj1199@hotmail.com (T.I.B.A.); hussienyazeed@yahoo.com (Y.A.E.); 6 Department of Microbiology, Faculty of Veterinary Medicine, Ankara University, Diskapi, Ankara 06110, Turkey; 7 Austrian Agency for Health and Food Safety (AGES), Spargelfeldstrasse 191, A-1220 Vienna, Austria; angelika.loitsch@ages.at; 8 UMR CIRAD INRA, Animal, Santé, Territoires, Risques et Ecosystèmes (ASTRE), CEDEX 05, 34398 Montpellier, France; adama.diallo@cirad.fr; 9 Laboratoire National d’Elevage et de Recherches Vétérinaires, Institut Sénégalais de Recherches Agricoles (ISRA), Dakar-Hann, Dakar BP 2057, Senegal

**Keywords:** LSDV, GPCR gene, RPO30 gene, EEV glycoprotein, B22R gene

## Abstract

Lumpy skin disease (LSD), an economically significant disease in cattle caused by lumpy skin disease virus (LSDV), is endemic to nearly all of Africa. Since 2012, LSDV has emerged as a significant epizootic pathogen given its rapid spread into new geographical locations outside Africa, including the Middle East, Eastern Europe, and Asia. To assess the genetic diversity of LSDVs in East Africa, we sequenced and analyzed the RPO30 and GPCR genes of LSDV in twenty-two archive samples collected in Ethiopia, Kenya, and Sudan before the appearance of LSD in the Middle East and its incursion into Europe. We compared them to publicly available sequences of LSDVs from the same region and those collected elsewhere. The results showed that the East African field isolates in this study were remarkably similar to each other and to previously sequenced field isolates of LSDV for the RPO30 and GPCR genes. The only exception was LSDV Embu/B338/2011, a field virus collected in Kenya, which displayed mixed features between the LSDV Neethling vaccine and field isolates. LSDV Embu/B338/2011 had the same 12-nucleotide insertion found in LSDV Neethling and KS-1 vaccines. Further analysis of the partial EEV glycoprotein, B22R, RNA helicase, virion core protein, NTPase, and N1R/p28-like protein genes showed that LSDV Embu/B338/2011 differs from previously described LSDV variants carrying the 12-nucleotide insertion in the GPCR gene. These findings highlight the importance of the constant monitoring of genetic variation among LSDV isolates.

## 1. Introduction

Lumpy skin disease (LSD) is a severe contagious disease in cattle, characterized by the appearance of nodules on the skin and enlarged superficial lymph nodes. The causative agent, lumpy skin disease virus (LSDV), belongs to the Capripoxvirus genus of the family Poxviridae [1,2]. LSDV has a double-stranded DNA genome of approximately 151 kb. It is closely related to sheep poxvirus (SPPV) and goat poxvirus (GTPV), two other members of the Capripoxvirus genus. However, the three viruses have several subtle genetic variations, causing differences in the virulence and host range of the capripoxviruses. These differences make LSDV a host-specific pathogen for cattle [3], although LSDV DNA was reported to be found in springbok antelopes in South Africa [4].

As LSD has a considerable economic impact on the cattle industry, the World Organization for Animal Health (OIE) has listed it as a notifiable disease [5]. In East Africa, Kenya was the first country to report LSD in 1957 [6], followed by Sudan in 1971 [7] and Ethiopia and Somalia in 1983 [8]. LSD is endemic to most African countries except Tunisia, Morocco, and Libya. The disease has also spread into most of the Middle East, Eastern Europe, and Asia [2,9,10,11,12,13,14]. East Africa has a large cattle population with important cross-border trade along the boundaries of Kenya, Ethiopia, Somalia, and Sudan. As this region is also a significant livestock export market for North Africa and the Arabian Peninsula, it was suggested that LSDV was re-introduced in Egypt in 2006 via infected cattle imported from the Horn of Africa [15]. This seems to have been the starting point before the disease spread further in the Middle East [16].

Therefore, this work focused on the molecular characterization of LSDV isolates collected in Kenya, Ethiopia, and Sudan before the wave of LSD in the Middle East, Europe, and Asia.

Molecular epidemiological studies of LSDV rely on the analysis of various regions of its genome, such as the GPCR, RPO30, and EEV genes [4,17,18,19]. Before identifying recombinant LSDVs and field-related LSDVs, GPCR and EEV were also suitable to differentiate LSDV vaccines from LSDV field strains.

Although LSD is endemic to East Africa, reports on the molecular characterization of LSDV in the region are inadequate. Previously, the GPCR and RPO30 genes of LSDV field isolates collected between 2008 and 2012 from several areas in Ethiopia were analyzed [18]. Our study further expands this analysis by investigating new outbreaks in Ethiopia and looking at isolates from Kenya and Sudan, where little insight was available on LSDV’s genomic data from post-2000 outbreaks. Because of the large size of the poxvirus genome, multiple-gene or whole-genome analysis is necessary to supplement the available molecular data.

The present study describes the comprehensive molecular characterization of LSDV isolates from East Africa.

## 2. Materials and Methods

### 2.1. Samples and DNA Extraction

**Figure 1:**
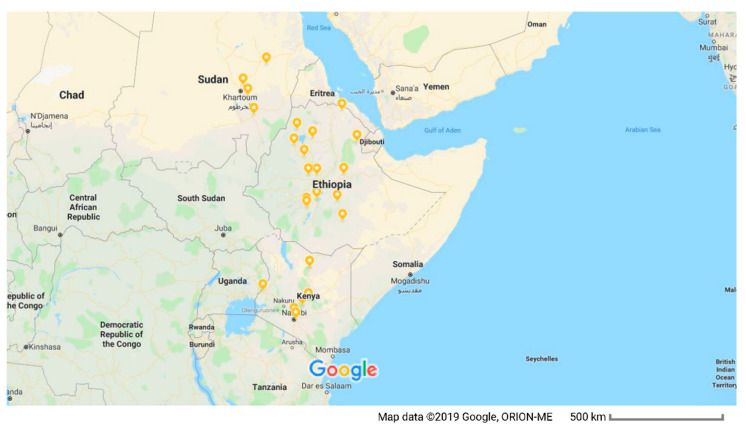
Map of East Africa showing Sudan, Kenya, and Ethiopia, and the approximate geographical origins (highlighted in orange) of LSDV isolates included in this study.

This study included 22 isolates (Table 1) of lumpy skin disease virus collected at various geographical locations in Ethiopia, Kenya, and Sudan (Figure 1). The total DNA was extracted from clinical samples and cell culture supernatants using the AllPrep DNA/RNA extraction kit (Qiagen, Hilden, Germany) according to the manufacturer’s instructions. The DNA was eluted using 80 μL elution buffer and stored at −20 °C until use.

### 2.2. Amplification of RPO30, GPCR, EEV Glycoprotein, and B22R Genes

For all 22 samples, RPO30 and GPCR were amplified as previously described [18]. A pair of primers, EEVGly F-5′-ATGGGAATAGTATCTGTTGTATACG-3′ and EEVGly R-5′-CGAACCCCTATTTACTTGAGAA-3′ [11], were used for the amplification of fragments containing the partial EEV glycoprotein (encoded by ORF LSDV126) and hypothetical protein LSDV 127 gene.

The PCR reaction was performed in a reaction volume of 20 μL containing 500 nM of each of the forward and reverse primers, 0.2 mM of dNTPs, 1× buffer (Qiagen), 2.5 U of Taq DNA polymerase (Qiagen), and 2 μL of template DNA. The thermal cycler (Bio-Rad, USA) parameters were: initial denaturation at 95 °C for 4 min, followed by 35 cycles at 95 °C for 40 s, 55 °C for 30 s, and 72 °C for 1 min, and a final extension step at 72 °C for 7 min. The partial B22R gene was amplified as described by [20].

The amplified PCR amplicons were separated by electrophoresis on a 1.5% agarose gel at 100 V for 60 min and visualized using a Gel Documentation System (Bio-Rad, Hercules, CA, USA).

### 2.3. Sequencing and Phylogenetic Analysis

The positive PCR products were purified using the Wizard SV Gel and PCR clean-up system kit (Promega, Dane County, WI, USA) according to the manufacturer’s instructions. The purified PCR products were sequenced at LGC Genomics (Berlin, Germany). The sequence data were assembled using Vector NTI 11.5 software (Invitrogen, Carlsbad, CA, USA). The generated sequences were submitted to GenBank under accession numbers MK302070 to MK302091 and MK302092 to MK302113 for the RPO30 and GPCR genes, respectively, and MN161848 for the partial EEV glycoprotein of LSDV Embu/B338/2011.

Nucleotide sequences were aligned using the MUSCLE algorithm and the codon option implemented in MEGA software version 7.0.26 [21]. The complete RPO30 and GPCR gene sequences of 11 additional LSDVs from East Africa (Table 1), 17 LSDVs from other regions, 7 GTPVs, and 4 SPPVs retrieved from GenBank were included for comparative analyses.

Bayesian phylogenetic inference was performed with BEAST v1.8.4 [22] using the HKY+G nucleotide substitution and a UPGMA starting tree option [22]. The Markov chain Monte Carlo method was run with BEAST for 10,000,000 generations, with a sample taken every 10,000 generations. The maximum clade credibility (MCC) was produced using TreeAnnotator after discarding the 10% burn-in determined using the TRACER program. The tree was visualized with the associated meta-data using the ggtree package in R [23]. A section of the alignment of the GPCR, between nucleotide positions 80 and 120, was visualized together with the GPCR tree [23].

### 2.4. Targeted Next-Generation Sequencing of Selected Variable Sites in the LSDV Genome

To further examine the differences between LSDV Embu and previously described LSDVs, and to rule out the possibility of a mixture between vaccine and field LSDV strains, we analyzed 7 selected hotspots using next-generation sequencing. These hotspots contain sites with nucleotide differences between the LSDV Neethling vaccine and field viruses or sites with nucleotide differences between the historical LSDVs from Kenya and vaccine-related field strains [24].

A PCR reaction was performed in a reaction volume of 20 μL containing 500 nM of each of the forward and reverse primers (Supplementary Table S1), 0.2 mM of dNTPs, 1× buffer (Qiagen), 2.5 U of Taq DNA polymerase (Qiagen), and 2 μL of template DNA. The thermal cycler (Bio-Rad, USA) parameters were: initial denaturation at 95 °C for 4 min, followed by 35 cycles at 95 °C for 30 s, 55 °C for 30 s, and 72 °C for 1 min, and a final extension step at 72 °C for 7 min. The positive PCRs were checked on a 1.5% (*w*/*v*) agarose gel. The amplicons were pooled and further purified using a 1.8X Agencourt AMPure XP kit (Beckman Coulter, Brea, CA, USA), and their concentrations were estimated using nanodrop ND-1000 Spectrophotometer (NanoDrop Technologies, Wilmington, DE, USA). Approximately 50–100 ng of the purified amplicons were enzymatically fragmented to 200 bp length using Ion Shear Plus reagents (Thermo Fisher Scientific, Waltham, MA, USA).

The sequencing library was prepared using an Ion Xpress™ Plus Fragment Library Kit (Thermo Fisher Scientific, Waltham, MA, USA) as per the manufacturer’s protocol and size-selected using Pippin Prep (Sage Science, Inc., Beverly, MA, USA). The libraries were clonally amplified and enriched using the Ion OneTouch system with the Ion 540™ Kit- OT2 reagents and Ion One Touch ES (enrichment system) as per the manufacturer’s instructions. Template-enriched ISPs were loaded onto the Ion540 chip and were sequenced with 500 flows to generate 200 bp reads on the Ion Torrent S5 sequencer (Thermo Fisher Scientific). The raw sequences were cleaned to remove low-quality and short reads using fastq-mcf v1.04.676 (ea-utils), and their quality was assessed with FastQC (v. 011.5). After mapping the cleaned raw reads against the reference sequence (LSDV NI-2490, NC_003027) using bowtie (v0.7.17), SAMtools (v1.11) was used to generate Mpileup files, and variant calling was performed using BCFtools (v1.9). The sorted reads were displayed within the Integrated Genome Viewer (IGV, v2.8.0) browser [25] to visualize possible mix populations at base mismatch sites (known to vary between the LSDV Neethling-related viruses and common LSDV field isolates). The consensus sequence for each targeted fragment was extracted using the IGV browser. Finally, the consensus sequences were compared to the corresponding fragments in publicly available complete genome sequences of LSDVs by multiple sequence alignments using BioEdit (v7.2.5).

## 3. Results

### 3.1. PCR Amplification and Sequencing

For each of the 22 samples, we successfully amplified and sequenced two fragments for the RPO30 gene (554 bp and 520 bp) and three for the GPCR gene (617 bp, 603 bp, and 684 bp) and assembled them to produce the complete RPO30 and GPCR gene sequences. We amplified and sequenced the partial EEV glycoprotein (931–958 bp) and the B22R (250 bp) genes of LSDV Embu/B338/2011.

### 3.2. Analysis of the RPO30 Gene

**Figure 2:**
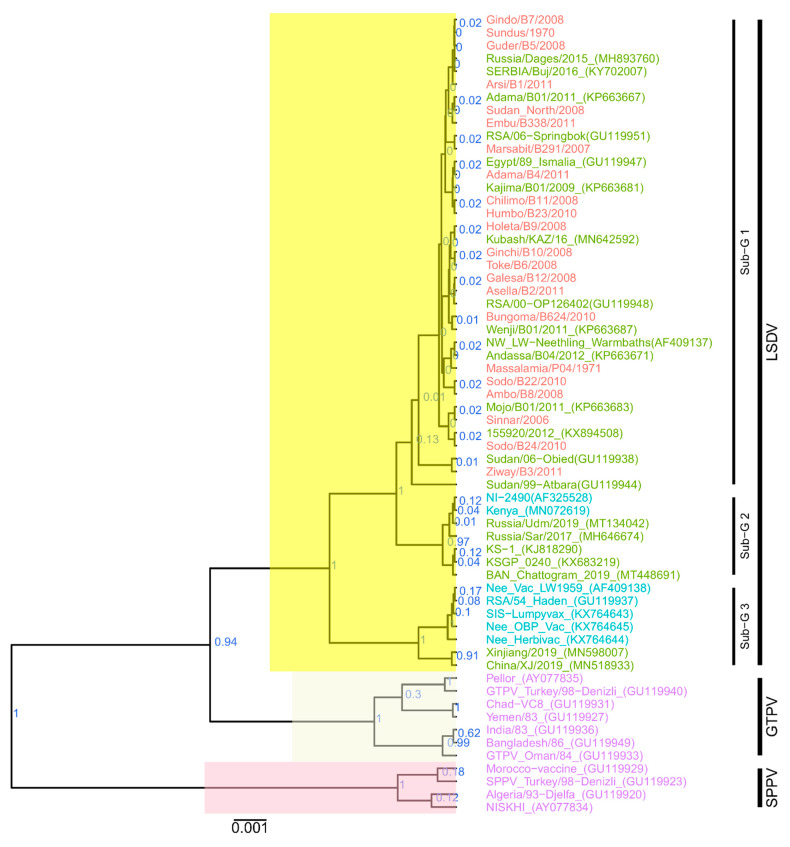
Maximum clade credibility (MCC) tree based on the complete RPO30 gene sequences of capripoxviruses. The posterior probabilities are plotted as respective node labels. The sequences of this study are highlighted in red, and reference sequences are represented with their accession numbers (blue for LSDVs collected before 1960 and green for those collected after 1960). SPPVs and GTPVs are shown in purple.

The phylogenetic tree based on the RPO30 gene showed that all 22 isolates in this study belong to LSDV. The phylogenetic reconstructions produced three subgroups for LSDVs with substantial posterior probabilities (Figure 2). Subgroup 1 included all the isolates in this study and common LSDV field isolates from East Africa, South Africa, North Africa, West Africa, and Europe. Subgroup 2 included mainly isolates from Kenya, such as LSDV NI-2490 (AF325528) and LSDV KSGP_O240 (KX683219); LSDV from Bangladesh; and two recombinant LSDV field isolates from Russia, LSDV Russia/Udmurtiya/2019 (MT134042) and LSDV Russia/Saratov/2017 (MH646674). Subgroup 3 included LSDV Neethling-derived vaccines and RSA/54 Haden (FJ869376), both from South Africa and recovered from outbreaks that occurred before 1960, and the LSDV field isolates from China (Figure 2).

Multiple sequence alignments of the RPO30 gene showed 100% identity among the East African isolates of this study, at both the nucleotide and amino acid levels, except for LSDV Bungoma/B624/2010. This isolate, collected in Kenya, presented a non-synonymous mutation (A/G, position 338), leading to an E to G amino acid change.

The comparison of the newly sequenced RPO30 genes with publicly available RPO30 sequences from East Africa showed high similarity between the sequences. LSDV KSGP_O240 (KX683219) and similar vaccines as well as LSDV NI-2490 (AF325528) and LSDV Kenya (MN072619), two field isolates from Kenya. Each presented a non-synonymous mutation, T/C (position 292), leading to an S to P change in their amino acid sequences. LSDV Sudan/06-Obied (GU119938) and LSDV Sudan/99-Atbara (GU119944), two previously sequenced isolates from Sudan, each presented one non-synonymous mutation, C/A (position 41) and A/G (position 305), respectively, leading to T to N and D to G amino acid changes, respectively.

### 3.3. Analysis of the GPCR Gene

**Figure 3:**
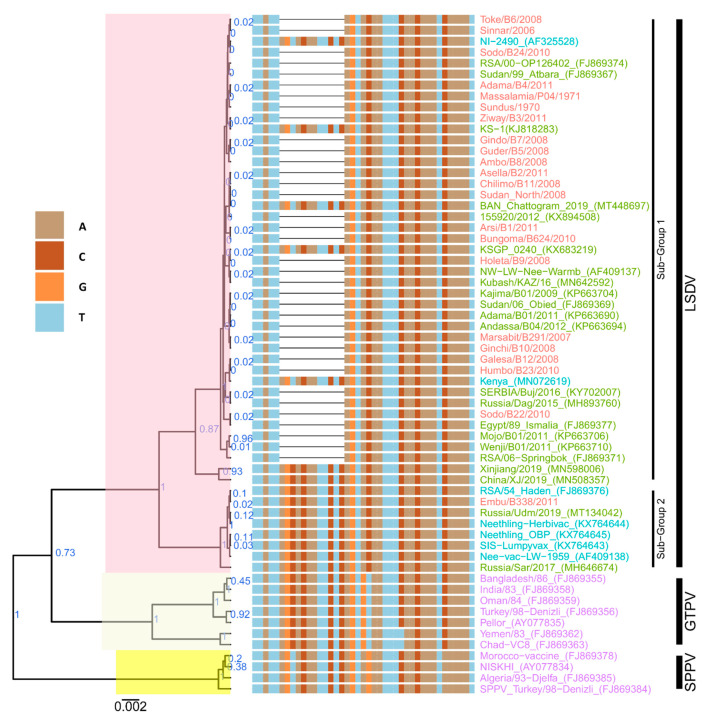
Maximum clade credibility (MCC) tree based on the complete GPCR gene sequences of capripoxviruses, plotted together with multiple sequence alignment. Only the portion of the alignment between positions 80 and 120 is shown. The posterior probabilities are plotted as respective node labels. Study sequences are highlighted in red and reference sequences are represented with their accession numbers (blue for LSDVs collected before 1960 and green for those collected after 1960). SPPVs and GTPVs are shown in purple.

As observed for the RPO30 gene, the phylogenetic tree of the GPCR genes also confirmed all 22 isolates in this study to be LSDVs. In contrast to the RPO30 tree, the GPCR tree produced only two subgroups with strong posterior probability values: 21 out of 22 LSDVs in this study clustered with subgroup 1, along with previously sequenced LSDV isolates from East Africa and other African countries; common LSDV field isolates from Europe; LSDVs from Bangladesh and China; and LSDV KSGP_O240 (KX683219), LSDV NI-2490 (AF325528), and LSDV Kenya (MN072619). Subgroup 2 contained the isolate LSDV Embu/B338/2011 from Kenya, together with LSDV Neethling vaccine LW-1959 (AF409138) and similar vaccines, LSDV RSA/54 Haden (FJ869376) from South Africa, and two recombinant LSDVs from Russia (Figure 3).

A close inspection of the multiple sequence alignments of GPCR nucleotide sequences showed that the GPCR genes of the isolates in this study were almost 100% similar to each other at both the nucleotide and amino acid levels, except for LSDV Embu/B338/2011. Expanding the comparison to previously sequenced LSDV isolates from East Africa, we observed that the GPCR gene sequences shared 100% identity, except for isolates LSDV Wenji/B01/2011 (KP663710) and LSDV Mojo/B02/2011 (KP663707) from Ethiopia, as well as LSDV KSGP_O240 (KX683219) and LSDV NI-2490 (AF325528) and LSDV Kenya (MN072619), two historical field isolates from Kenya.

LSDV Wenji/B01/2011 differed from other East African field LSDVs with a T/C mutation (position 908 in the GPCR gene of common LSDV field isolates), leading to an F to P change in the amino acid sequence. There was also one synonymous nucleotide change each in LSDV Wenji/B01/2011 (KP663710) and LSDV Mojo/B02/2011 (KP663707). LSDV Embu/B338/2011 contained an insertion of 12 nucleotides (Figure 3), corresponding to amino acids I, L, S, and T, and was 100% identical to LSDV Neethling vaccine LW-1959 (AF409138) in the GPCR gene. The 12-nucleotide insertion was also present in LSDV KSGP_O240 (KX683219), two historical field isolates from Kenya (LSDV NI-2490 (AF325528) and LSDV Kenya (MN072619)), LSDVs from Bangladesh, LSDV RSA/54 Haden (GU119937) from South Africa, recombinant LSDVs from Russia (LSDV Russia/Udmurtiya/2019, LSDV Russia/Saratov/2017, and LSDV Dergachevskyi), and recent LSDV isolates from China (Figure 3). LSDV KSGP_O240 (KX683219) and the historical Kenyan isolates (LSDV Kenya and LSDV NI-2490) differed from LSDV Embu/B338/2011 and LSDV Neethling vaccine by 23 additional nucleotides, leading to four further amino acid changes (S/N, M/I, L/I, M/T).

### 3.4. Analysis of the EEV Glycoprotein and the B22R Genes of LSDV Embu/B338/2011

**Figure 4:**
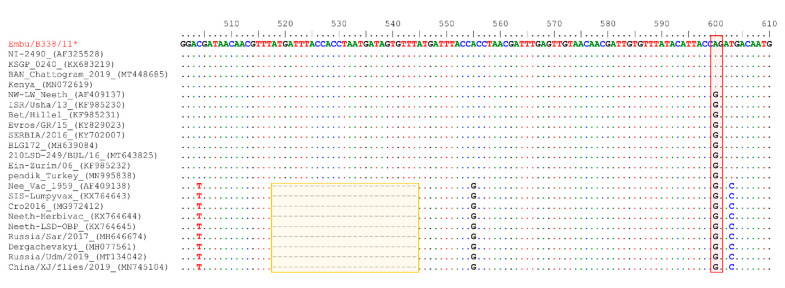
Multiple sequence alignments of the partial EEV glycoprotein nucleotide sequences of LSDV Embu/B338/2011 (highlighted in red, with a star after the name) with sequences of 22 additional LSDVs retrieved from GenBank. A unique sequence signature of 27 nucleotides only in LSDV Neethling-like viruses is highlighted in the box. Note also the A to G change highlighted the red box. Identical nucleotides are indicated with dots.

As the LSDV Embu/B338/2011 showed significant similarity to LSDV vaccines on the GPCR gene and LSDV field isolates on the RPO30 gene, we sequenced the EEV glycoprotein gene. We compared the results to the publicly available sequences for further elucidation. The EEV glycoprotein gene sequence of LSDV Embu/B338/2011 contained an insertion of 27 nucleotides (9 amino acids), as previously reported in LSDV field isolates; LSDV KSGP-0240-derived vaccines; LSDVs from Bangladesh; and two historical LSDVs, LSDV NI2490 (1958) and LSDV Kenya (1950), both from Kenya. This fragment was absent in LSDV Neethling vaccine-like viruses, LSDVs from China, and the recombinant LSDVs described in Russia (Figure 4).

**Figure 5:**
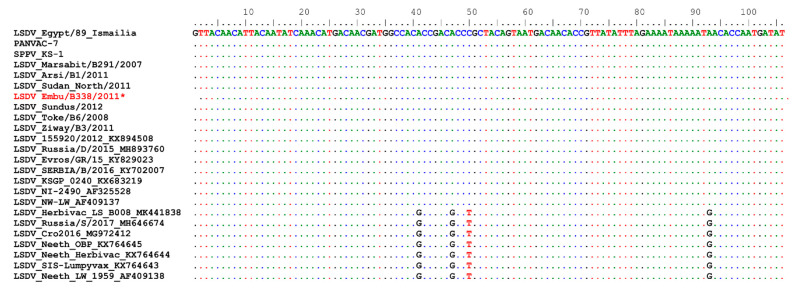
Multiple sequence alignments of the partial B22R gene sequences of LSDV Embu/B338/2011 (highlighted in red, with a star after the name) and 23 additional LSDVs retrieved from GenBank. The variable sites appear as letters, and identical nucleotides appear as dots.

This result confirmed that LSDV Embu/B338/2011 differed from the LSDV Neethling vaccine. We also sequenced the B22R gene of LSDV Embu/B338/2011 and compared it with publicly available LSDV sequences. The results showed that part of the B22R gene of LSDV Embu/B338/2011 was identical to that of field isolates and contained four mutations (A/G, A/G, C/T, A/G) compared with LSDV Neethling vaccine and LSDV Russia/Saratov/2017, a recombinant-like virus from Russia (Figure 5).

### 3.5. Analysis of Additional Variable Sites in the LSDV Genome by Targeted Next-Generation Sequencing

The comparative analysis of the partial sequences of the RNA helicase gene (LSDV049), virion core protein p4b gene (LSDV094), the NTPase (LSDV083) gene, and the N1R/p28-like protein gene (LSDV140), derived from the mapping of the NGS reads, revealed additional differences between LSDV Embu/B338/2011 and previously described LSDVs (Supplementary Table S2). There were three synonymous changes on the RNA helicase gene (C/T, A/G, and A/G at positions corresponding to 44,018, 44,168, and 44,202, respectively, in NC_003027) and another single synonymous mutation on the NTPase gene (A/T at the position corresponding to 75,218 in NC_003027) between LSDV Embu/B338/2011 and the two recombinant LSDVs from Russia (LSDV Saratov/2017 and LSDV Russia/Udm/2019). Similarly, we also found noticeable differences between LSDV Embu/B338/2011 and the South African vaccine-related field LSDVs. Hence, two synonymous nucleotide changes occurred on the NTPase gene (A/G and G/A at positions corresponding to 75,253 and 75,325, respectively, in NC_003027) and five occurred on the N1R/p28-like protein gene (A/G, G/A, G/T, A/T, and G/A, at positions corresponding to 133,035, 133,056, 133,108, 133,126, and 133,137, respectively, in NC_003027).

The visualization of the mapped and sorted reads for selected hotspots (Supplementary Figure S1) showed only one virus variant at each variable site. This indicates that only one viral population was present in the sample; therefore, LSDV Embu/B338/2011 is a new LSDV variant, not a mixture of two viruses.

## 4. Discussion

In this study, we analyzed LSDV isolates collected in East Africa before the appearance of LSD in the Middle East and its incursion into Europe. The East African LSDV field isolates covered in this study (newly sequenced and recovered from public databases) were collected between 1950 and 2012. Overall, except for LSDV Embu/B338/2011, the East African field isolates in this study were highly similar to each other and to the previously sequenced East African field isolates of LSDV for the RPO30 and the GPCR genes. Likewise, the East African isolates were also highly similar to LSDV field isolates encountered elsewhere, including the Middle Eastern and European isolates. As the LSDV genome is stable, limited variability is likely to have occurred following the escape of the virus from Africa to the Middle East and then to Europe.

The only exceptions were LSDV Embu/B338/2011, collected in 2011 in Kenya, which differed from other field isolates by an insertion of 12 nucleotides in its GPCR gene; this was previously described for several historical isolates, namely LSDV Neethling vaccine LW-1959 (AF409138), LSDV RSA/54 Haden (FJ869376), LSDV NI-2490 (AF325528) [3], LSDV Kenya (MN072619), LSDVs from Bangladesh [11], recent LSDV from China, and recombinant field LSDVs from Russia [26,27,28].

In contrast to our findings on the GPCR gene, LSDV Embu/B338/2011 was identical to LSDV field isolates and LSDV KSGP_O240 (KX683219) for the full RP30 gene, the partial EEV glycoprotein gene, and the partial B22R gene. Altogether, the analysis of these four genes showed that LSDV Embu/B338/2011 differs from the two commonly used LSDV vaccines in East Africa: the LSDV KSGP_O240 (KX683219)-derived vaccines and the LSDV Neethling vaccine.

Based on the full GPCR and RPO30 genes and the partial EEV glycoprotein and B22R genes, LSDV Embu/B338/2011 appears to be a mix between an LSDV field isolate and the LSDV Neethling vaccine. In a previous study, based on a comparative analysis of detailed physical maps of the genomes of four Capripoxvirus isolates, the authors suggested that the isolate Yemen goat-1, with mixed features of the Kenya cattle-1 Capripoxvirus field strain and the Iraq goat-1 vaccine strain, was a recombinant virus [29]. More recently, some reports highlighted the presence of recombinant field LSDVs in Russia [26,28]. Analyzing the full genome of LSDV Russia/Saratov/2017 collected at the Russian border with Kazakhstan, the authors suggested that this virus was a recombinant escape of one of the LSDV vaccines derived from the Neethling vaccine strain and a field isolate [26]. Interestingly, the comparison of the EEV glycoprotein and the B22R genes of LSDV Embu/B338/2011 to that of the Russian recombinant-like isolates LSDV Russia/Saratov/2017 suggests that they are different. While LSDV RUSSIA/Saratov/2017 resembled LSDV Neethling vaccine on the EEV glycoprotein and the B22R genes, LSDV Embu/B338/2011 was more related to field isolates on the same genes. This was further confirmed by the presence of additional variable sites on the RNA helicase and the NTPase genes of the sequences derived from the targeted NGS. Our results also highlighted some differences between LSDV Embu/B338/2011 and the recently sequenced vaccine-related field viruses from South Africa [24].

The epidemiological data could support the hypothesis that LSDV Embu/B338/2011 arose through recombination; however, because the isolate was from a previously vaccinated herd, it is also possible that such an isolate was circulating before the vaccination. For instance, LSDV NI-2490 and LSDV Kenya, two historical field isolates collected in Kenya in 1950 and 1958, respectively, also contain a 12-nucleotide insertion in the GPCR gene [4,30].

Several reports have suggested that the occurrences of LSDV in vaccinated flocks were because of vaccination failures or the potential residual virulence of vaccines such as LSDV KS1 [18,31,32,33]. However, our data show that LSDV KSGP O240 and LSDV Embu/B338/2011 are genetically different.

It is also interesting to note that LSDV isolates carrying the 12-nucleotide insertion in their GPCR gene were previously rare. For instance, LSDV Kenya occurred in 1950, followed by LSDV RSA/54 in 1954, LSDV NI-2490 in 1958, LSDV Neethling vaccine LW-1959 in 1958 and 1959, LSDV KS-1 in 1976, and the LSDV Embu/B338/2011 of this study in 2011. However, following the detection of recombinant LSDV RUSSIA/Saratov/2017 in 2017 and LSDV Russia/Udmurtiya/2019, all the new outbreaks characterized so far in Asia involved LSDV field isolates carrying the 12-nucleotide insertion in their GPCR gene. Additionally, the sequencing of archive samples showed that most vaccine-related field viruses from South Africa carry this 12-nucleotide insertion [24].

## 5. Conclusions

Our data suggest that LSDV genomes were remarkably stable over decades in East Africa; however, LSDV variants such as those with this 12-nucleotide insertion in their GPCR gene were circulating without being readily noticed.

These findings on LSDV Embu/B338/2011 highlight the importance of constant monitoring of LSDV genetics in the field. They also show the importance of analyzing multiple genes while addressing the differentiation between vaccines and field isolates.

## Figures and Tables

**Table 1:** LSDV isolates from East Africa included in this study and previous isolates.

No.	Strain Name	Host	Origin	Sample Type	Vaccination History	Year	Accession Number	
							RPO30	GPCR
1	* Marsabit/B291/2007	Cattle	Kenya	Skin lesion	Non-vaccinated	2007	MK302092	MK302070
2	* Embu/B338/2011	Cattle	Kenya	Skin lesion	Vaccinated	2011	MK302093	MK302071
3	* Bungoma/B624/2010	Cattle	Kenya	Skin lesion	Non-vaccinated	2010	MK302094	MK302072
4	* Arsi/B1/2011	Cattle	Ethiopia	Skin lesion	Non-vaccinated	2011	MK302095	MK302073
5	* Asella/B2/2011	Cattle	Ethiopia	Skin lesion	Non-vaccinated	2011	MK302096	MK302074
6	* Ziway/B3/2011	Cattle	Ethiopia	Skin lesion	Non-vaccinated	2011	MK302097	MK302075
7	* Adama/B4/2011	Cattle	Ethiopia	Skin lesion	Non-vaccinated	2011	MK302098	MK302076
8	* Guder/B5/2008	Cattle	Ethiopia	Skin lesion	Non-vaccinated	2008	MK302099	MK302077
9	* Sodo/B22/2010	Cattle	Ethiopia	Skin lesion	Non-vaccinated	2010	MK302100	MK302078
10	* Humbo/B23/2010	Cattle	Ethiopia	Skin lesion	Non-vaccinated	2010	MK302101	MK302079
11	* Sodo/B24/2010	Cattle	Ethiopia	Skin lesion	Non-vaccinated	2010	MK302102	MK302080
12	* Sundus/1970	Cattle	Sudan	Skin lesion	Non-vaccinated	1971	MK302103	MK302081
13	* Sudan North/2008	Cattle	Sudan	Skin lesion	Non-vaccinated	2008	MK302104	MK302082
14	* Sinnar/2006	Cattle	Sudan	Skin lesion	Non-vaccinated	2006	MK302105	MK302083
15	* Toke/B6/2008	Cattle	Ethiopia	Swab samples	Non-vaccinated	2008	MK302106	MK302084
16	* Gindo/B7/2008	Cattle	Ethiopia	Swab samples	Non-vaccinated	2008	MK302107	MK302085
17	* Ambo/B8/2008	Cattle	Ethiopia	Swab samples	Non-vaccinated	2008	MK302108	MK302086
18	* Holeta/B9/2008	Cattle	Ethiopia	Swab samples	Non-vaccinated	2008	MK302109	MK302087
19	* Ginchi/B10/2008	Cattle	Ethiopia	Swab samples	Non-vaccinated	2008	MK302110	MK302088
20	* Chilimo/B11/2008	Cattle	Ethiopia	Swab samples	Non-vaccinated	2008	MK302111	MK302089
21	* Galesa/B12/2008	Cattle	Ethiopia	Swab samples	Non-vaccinated	2008	MK302112	MK302090
22	* Massalamia/P04/1971	Cattle	Sudan	Cell culture	Non-vaccinated	1971	MK302113	MK302091
23	Sudan/99-Atbara	Cattle	Sudan			1999	GU119944	FJ869367
24	Sudan/06-Obied	Cattle	Sudan			2006	GU119938	FJ869369
25	KSGP 0240	Sheep	Kenya			1976	KX683219	KX683219
26	KS-1	Sheep	Kenya			1976	KJ818290	KJ818283
27	NI-2490	Cattle	Kenya			1958	AF325528	AF325528
28	Kenya	Cattle	Kenya			1950	MN072619	MN072619
29	Adama/B01/2011	Cattle	Ethiopia			2011	KP663667	KP663690
30	Andassa/B04/2012	Cattle	Ethiopia			2012	KP663671	KP663694
31	Kadjima/B01/2009	Cattle	Ethiopia			2009	KP663681	KP663704
32	Mojo/B01/2011	Cattle	Ethiopia			2011	KP663683	KP663706
33	Wenji/B01/2011	Cattle	Ethiopia			2011	KP663687	KP663710

* LSDV sequenced for this study.

## Data Availability

The data that support the findings of this study are openly available in NCBI at https://www.ncbi.nlm.nih.gov/nuccore/ (accessed on 25 May 2021).

## Supplementary Materials

The following are available online at https://www.mdpi.com/article/10.3390/microorganisms9061142/s1, Figure S1: Visualization of targeted next-generation sequencing reads for four selected genes in LSDV Embu/B338/2011. The reads were mapped against the LSDV NI-2490 (NC_003027), sorted, and displayed in the IGV browser. The blue arrows indicate the positions of the variable sites in comparison to the reference. Note the absence of subpopulation (only one color per variable site), Table S1: Primers used for targeted sequencing by NGS. The primer sequences, the amplicon size and their positions in LSDV NI-2490 (NC_003027) and the targeted genes are provided, Table S2: Nucleotides polymorphism identified between representative LSDVs for vaccine strains (AF409138 and KX683219), field strains (KY829023 and NC_003027), recombinant viruses (MH646674 and MT134042), and vaccine-related field viruses (MN636841). The polymorphic nucleotides in four targeted genes and their position in LSDV NI-2490 (NC_003027) are shown. Nucleotide changes in LSDV Embu/B338/2011 compared to recombinant viruses (MH646674 and MT134042) are highlighted in yellow. The differences to the South African vaccine-related field virus are highlighted in green.
