# Supplementary material for: Molecular Analysis of East African Lumpy Skin Disease Viruses Reveals a Mixed Isolate with Features of Both Vaccine and Field Isolates
Source: Microorganisms. 2021 May 26;9(6):1142. doi: 10.3390/microorganisms9061142 (PMC8229927; doi:10.3390/microorganisms9061142)
Supplement: Supplementary file 1 [file microorganisms-09-01142-s001.zip › supplementary files/Supplement Figure S1.pdf]

Supplement Figure S1. Visualization of targeted next-generation sequencing reads for four selected genes in LSDV Embu/B338/2011. The reads were mapped against the LSDV NI-2490 (NC_003027), sorted, and displayed in the IGV browser. The blue arrows indicate the positions of the variable sites in comparison to the reference. Note the absence of subpopulation (only one color per variable site).
