# Supplementary material for: Molecular Analysis of East African Lumpy Skin Disease Viruses Reveals a Mixed Isolate with Features of Both Vaccine and Field Isolates
Source: Microorganisms. 2021 May 26;9(6):1142. doi: 10.3390/microorganisms9061142 (PMC8229927; doi:10.3390/microorganisms9061142)
Supplement: Supplementary file 1 [file microorganisms-09-01142-s001.zip › supplementary files/Supplement Table S1.pdf]

Supplement Table S1. Primers used for targeted sequencing by NGS. The primer sequences, amplicon sizes and their positions in LSDV NI-2490 (NC_003027) and the targeted genes are provided.

**Primer name**	**sequence**	**Product size/targeted gene (ORF)/position in LSDV NI-2490**
EEV_GP_CaPVFw	ATACGTCGTAGTACCATTTTCATTT	505 pb/ EEV glycoprotein gene (LSDV126/(116161- 116665)
EEV_GP_CaPVRv	TGACTCGGAATTATTTTTGTGC	
B22R_CaPVFw	TCATTTTCTTCTAGTTCCGACGA	863 bp/ B22R-like protein gene (LSDV 134)/124207-125069)
B22R_CaPVRv	TTCGTTGATGATAAATAACTGGAAA	
GPCR_div_CaPVFw	CAGTTAGTAGCGCCACCATGTA	414 bp/ G protein-coupled chemokine receptor-like protein gene (LSDV011)/(7686- 8099)
GPCR_div_CaPVRv	AAGGAAACACCAACACGAAAA	
R-Hel_CaPVFw	ACAGCGACACTAGAGGACGA	400 bp/ RNA-Helicase gene (LSDV049)/(43968- 44367)
R-Hel_CaPVRv	TCGTCACGCTTGATTCTAAA	
NTP_CaPVFw	CATTGGGAAGAAGGATTTTG	386 bp/ NTPase gene (LSDV083)/ (75005-75390)
NTP_CaPVRv	CCACATTCCCGAGAAGAAAA	
4b_CaPVFw	GCTCTTAACAGACATGCTGGAG	360 bp/ virion core protein p4b gene (LSDV094)/(88690- 89049)
4b_CaPVRv	ATCTCAGCAGGGGCTAGACA	
E3_CaPVFw	TTATACGCGTCTACCGAAAGAA	302 bp/E3 ubiquitin-protein ligase p28-like protein gene (LSDV140)/(133069 133371)
E3_CaPVRv	GGAATTCATTCCTACATACTGGACA	
