# Supplementary material for: Molecular Analysis of East African Lumpy Skin Disease Viruses Reveals a Mixed Isolate with Features of Both Vaccine and Field Isolates
Source: Microorganisms. 2021 May 26;9(6):1142. doi: 10.3390/microorganisms9061142 (PMC8229927; doi:10.3390/microorganisms9061142)
Supplement: Supplementary file 1 [file microorganisms-09-01142-s001.zip › supplementary files/Supplement Table S2.pdf]

Supplement Table S2. Nucleotides polymorphism identified between representative LSDVs for vaccine strains (AF409138 and KX683219), field strains (KY829023 and NC_003027), recombinant viruses (MH646674 and MT134042), and vaccine-related field viruses (MN636841). The polymorphic nucleotides in four targeted genes and their position in LSDV NI-2490 (NC_003027) are shown. Nucleotide changes in LSDV Embu/B338/2011 compared to recombinant viruses (MH646674 and MT134042) are highlighted in yellow. The differences to the South African vaccine-related field virus are highlighted in green.

Position in LSDV NI_2490	NI_2490 (NC_003027)	Embu/B338/2011	Russia/Saratov/2017 (MH646674)	Russia/Udm/2019 (MT134042)	Evros/GR/15 (KY829023)	KSGP 0240 (KX683219)	Neethling vaccine LW 1959 (AF409138)	220-1-NW-RSA-1993 (MN636841)	Gene
44018	T	C	T	T	T	T	C	C	RNA-Helicase gene (LSDV049)
44168	G	A	G	G	G	G	A	A	
44202	G	A	G	G	G	G	A	A	
75080	A	G	G	A	A	A	G	G	NTPase gene (LSDV083)
75218	T	A	T	T	T	T	A	A	
75253	A	A	G	A	A	A	G	G	
75325	G	G	G	G	A	G	A	A	
88924	C	T	T	T	C	C	T	T	virion core protein p4b gene (LSDV094)
88938	C	T	T	T	C	C	T	T	
88969	C	A	A	A	C	C	A	A	
88970	G	G	G	G	A	G	G	G	
88975	C	T	T	T	T	C	T	T	
133019	C	C	C	C	T	C	C	C	E3 ubiquitin-protein ligase p28-like protein gene (LSDV140)
133035	A	A	A	A	A	A	G	G	
133056	G	G	G	G	G	G	A	A	
133108	G	G	G	G	G	G	T	T	
133126	A	A	A	A	A	A	T	T	
133137	G	G	G	G	G	G	A	A	
